# T-helper 17 lymphocytes in ocular cicatricial pemphigoid

**Published:** 2009-07-28

**Authors:** Alessandro Lambiase, Alessandra Micera, Flavio Mantelli, Caterina Moretti, Antonio Di Zazzo, Eleonora Perrella, Sergio Bonini, Stefano Bonini

**Affiliations:** 1 Interdisciplinary Center for Biomedical Research (CIR), Laboratory of Ophthalmology, University of Rome “Campus Bio-Medico,” Rome, Italy; 2 G.B. Bietti Eye Foundation, Rome, Italy; 3 Pathology, University of Rome “Campus Bio-Medico,” Rome, Italy; 4 Internal Medicine, Second University of Naples, Naples, Italy

## Abstract

**Purpose:**

T-helper 17 lymphocytes (Th17) were identified in the healthy conjunctiva and in patients with ocular cicatricial pemphigoid (OCP), a disease characterized by chronic ocular surface inflammation.

**Methods:**

Conjunctival biopsies and blood samples were obtained from 10 patients with OCP (4 males, 6 females; 57–90 years of age) and 6 age/sex matched healthy subjects. Conjunctival samples were immunostained with anti-human IL17/CD4 antibodies and stained cells were then counted by confocal microscopy in three 60X field images per each sample. Mononuclear cells were isolated from both OCP and healthy blood samples and evaluated for IL17 and CD4 by FACS. IL17, TGF-β, IL4, and IFN-γ levels were determined in plasma of OCP and healthy patients by ELISA.

**Results:**

The presence of Th17 lymphocytes in conjunctival biopsies was significantly (p<0.05) increased in patients with OCP (14.9±12.8 cells per microscopic field) compared to healthy subjects (0.5±0.8 cells per microscopic field). Th17 lymphocytes comprised 72% of CD4^+^ cells in four stage-III OCP conjunctival samples. No significant difference was observed for IL17 in peripheral blood of OCP versus healthy subjects.

**Conclusions:**

In this study, we report an increased localization of Th17 lymphocytes in OCP conjunctiva, not accompanied by similar findings in peripheral blood. This finding suggests an increased recruitment of Th17 lymphocytes in conjunctiva and/or a dysfunctional local immune response in the chronically inflamed conjunctiva of OCP. Our findings are in line with previously reported evidence demonstrating that Th17 cells play a critical pathogenic role in mucosal autoimmunity.

## Introduction

The family of CD4^+^ T-cells includes different subtypes of T-helper (Th) lymphocytes characterized by specific cytokine profiles: Th1 cells secrete IFNγ and IL-2; Th2 cells secrete IL-4, IL-5, and IL-13; and Th17 cells secrete IL-17 [1-5]. Recently, Th17 lymphocytes have been characterized as potent inducers of tissue inflammation in several autoimmune diseases, such as inflammatory bowel disease (IBD), psoriasis, multiple sclerosis (MS), lupus erythematosus systemicus, rheumatoid arthritis (RA), and Bechet’s disease, through the activation of a wide range of inflammatory mediators (IL-6 and IL-8), angiogenesis, and the induction of immune cell activation, particularly neutrophils [6-15]. In fact, increased levels of IL-17 have been detected in biopsies of skin from psoriasis patients, of gut from IBD patients, of brain from MS patients, and of the synovium as well as synovial fluid from RA patients [16]. IL-17 was in fact associated with an increase in both activity and severity of these diseases [17,18]. Concordant results have been obtained in mouse models of RA, in which the local release of IL-17 induces massive damage with extensive inflammatory cell migration, bone erosions, and cartilage degradation [19].

These data indicate a pivotal role of Th17 in regulating the mucosal immune response through migration of immune cells to target organs and induction of pro-inflammatory cytokine release, ultimately leading to tissue damage. However, to date the role of Th17 in ocular inflammatory diseases has been studied only in uveitis and scleritis [12,20-22].

In this study, we investigated the role played by Th17 lymphocytes in ocular cicatricial pemphigoid (OCP), an autoimmune disease characterized by chronic mucosal inflammation with T-cell dysfunction and infiltration of immune cells in the conjunctiva [23-25].

## Methods

### Patients and biological sample handling

The study was performed in accordance to the tenets of the Declaration of Helsinki for research involving human subjects and the Intramural Ethics Committee approved the project. Informed consent was signed by each participant.

**Figure 1:**
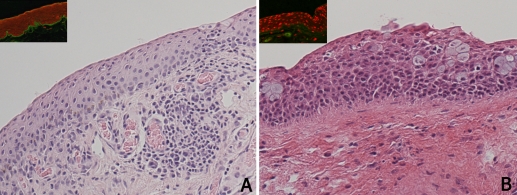
Histology of OCP conjunctiva. OCP conjunctiva was infiltrated with immune cells, including lymphocytes, plasma cells and leukocytes (**A**), whereas healthy conjunctiva was free of immune cells (**B**). A linear direct immunofluorescence labeling (green) of autoantibodies in the conjunctival basal membrane was observed in all OCP samples (insert **A**). No immunostaining was observed in control samples (insert **B**).

Biopsies from temporal bulbar conjunctiva and peripheral blood samples were obtained from 10 patients with OCP (4 males, 6 females; 57–90 years of age) and 6 age/sex matched healthy subjects with no history of dry eye or other ocular diseases at the time of cataract surgery. Healthy subjects were carefully screened for dry eye by rose bengal/fluorescein staining and lacrimal functional tests including tear osmolarity, Schirmer tests, and Tear Film Break-up time test. Table 1 shows the main clinical characteristics of the OCP patients recruited for this study at the time of the first visit to our Cornea and External Eye Disease Unit (Campus Bio-Medico University of Rome, Italy). The diagnosis of OCP was based on history, clinical diagnosis, and specific linear direct immunofluorescent labeling of conjunctival basal membrane (Figure 1) [26]. The stage of disease was defined according to the Foster-Mondino classification [27,28]: eight OCP patients recruited for this study were in stage III, one patient was in stage II and one in stage I (Table 1).

**Table 1:** Main clinical characteristics of the OCP patients recruited for this study.

**ID**	**Age**	**Gender**	**Foster classification [**29,30**]**	**Cornea**	**Schirmer test I mm/5 min**	**Disease activity**	**Topical therapy**
1	90	M	III	SPK	2	quiescent	steroids
2	49	F	III	SPK	4	active	artificial tears
3	57	F	III	band keratopathy	9	active	artificial tears
4	69	M	III	leucoma	5	quiescent	artificial tears
5	60	M	II	leucoma, SPK	1	active	artificial tears
6	63	F	I	SPK	2	quiescent	artificial tears
7	76	F	III	SPK	5	quiescent	artificial tears
8	83	M	III	SPK	4	active	steroids
9	79	F	III	band keratopathy	0	quiescent	artificial tears, steroids
10	68	F	III	SPK	2	active	artificial tears

In the table, SPK indicates superficial punctate keratitis.

Each conjunctival biopsy was 10% formalin fixed and processed for light (histology) or confocal microscopy (immunofluorescence). Blood samples (10 ml) were collected in heparinized vacutainer vials and processed for flow cytometry (enriched peripheral blood mononuclear cells) and ELISA (plasma).

All analytical grade reagents and plasticware were purchased from SERVA (Weidelberg, Germany) and NUNC (Roskilde, Denmark), unless specified differently in the text.

### Light and confocal microscopy

Paraffin-embedded conjunctival biopsies were serially sliced into 5 μm sections, attached to pretreated slides (Bioptica, Milan, Italy) and processed for either basic histology (hematoxylin and eosin; Bioptica) or direct immunofluorescence, according to standardized procedures.

Direct immunofluorescence on conjunctival biopsies was performed to demonstrate OCP conjunctival basal membrane positivity to goat anti-human FITC-conjugated immunoglobulin (AbD Serotec, Oxford, UK). To identify IL-17 and CD4 positive cells, immunofluorescence staining was performed. Endogenous peroxidase signal was quenched by incubation in 3% H_2_O_2_ for 5 min and 50 mM NH_4_Cl for 2min; antigen was then retrieved by enzymatic pretreatment with hyaluronidase (1mg/ml, in sodium acetic acid; ICN, Costa Mesa, CA). A brief blocking/permeabilizing step was performed for 20 min (0.8% BSA, BSA-0.3% Triton X-100 [TX] in 10 mM phosphate-buffered saline, PBS) before addition of the specific PE conjugated antihuman IL-17 antibodies (2 μg/ml; eBioscience, San Diego, CA), PE-Cy5.5-conjugated antihuman CD4 antibodies (2 μg/ml), or irrelevant isotype-matched IgG antibodies (eBioscience), as negative controls for staining specificity, diluted in 0.1% Tween-20-PBS. Sections of human nasal turbinates were used as positive controls for the internal procedure (data not shown) [29]. Antifade/gel (Vector, Burlingame, CA) mounted sections were examined at 60X magnification in oil immersion using an E2000U three-laser confocal microscope equipped with C1 software (Nikon, Tokjo, Japan).

Three optic fields per each section (3 slides per patient) were acquired randomly in a masked fashion by an independent observer and digitalized using Adobe Photoshop 7.0 (Adobe, San Jose, CA) software. Cell counts were performed by two independent observers. Each observer was blinded to the decision of the other observer [30-32].

### Flow cytometry

Peripheral blood mononuclear cells (PBMNCs) of OCP patients and controls were obtained from heparinized-whole blood samples, following gradient selection (lympholyteTM; Euroclone, Milan, Italy), according to a standardized procedure. Red cells were lysed (RBC Gentra solution; Qiagen, Mila, Italy) and white cells were directly stored in liquid nitrogen for the following experiments. Direct immunostaining was performed with phycoerythrin (PE)-conjugated antihuman IL17A (0.25 µg/106 cells; ebioscience) and PE-Cy5-conjugated antihuman CD4 (both at 0.25 µg/106 cells; ebioscience), according to the manufacturer's instructions. All samples were analyzed through a FACSCalibur flow cytometer (Becton Dickinson, Milan, Italy) equipped with an argon laser emitting at 488 nm and three band-pass detectors. Forward and side scatter signals were collected as linear signals, and all emissions were evaluated on a four-decade logarithmic scale. PE and PE-Cy5.5 signals were measured, respectively at 575 nm and 670 nm. Spectral overlap was minimized by electronic compensation before each series. CellQuest® software (Becton Dickinson) was used to acquire and evaluate 5,000 events with preserved side scatter signals and high membrane staining for specific antibodies [33]. Data were expressed as % of positive cells or mean fluorescence intensity, calculated as follows: ΔMFI=(specific MFI-aspecific MFI)/aspecific MFI.

### ELISA assay

To evaluate IL-17, TGF-β, IL-4, and IFN-γ in plasma collected from heparinized peripheral blood of OCP patients and controls, we performed a two-site ELISA (sensitivity=0.5 pg/ml) using commercially available kits and following the manufacturer’s instructions (eBioscience). Optical density (OD) was measured at λ450–550 by a microplate ELISA reader (Sunrise; Tecan Systems, Inc., San Jose, CA).

### Statistical analysis

All experiments were performed in duplicate and results are expressed as the mean±standard deviation of the mean (SD). Statistical analyses were performed using ANOVA followed by Tukey-Kramer post-hoc calculations. Analyses were performed using the statistical package StatView II for PC (Abacus Concepts Inc., Berkley, CA).A p value less than 0.05 was considered statistically significant.

## Results

**Figure 2:**
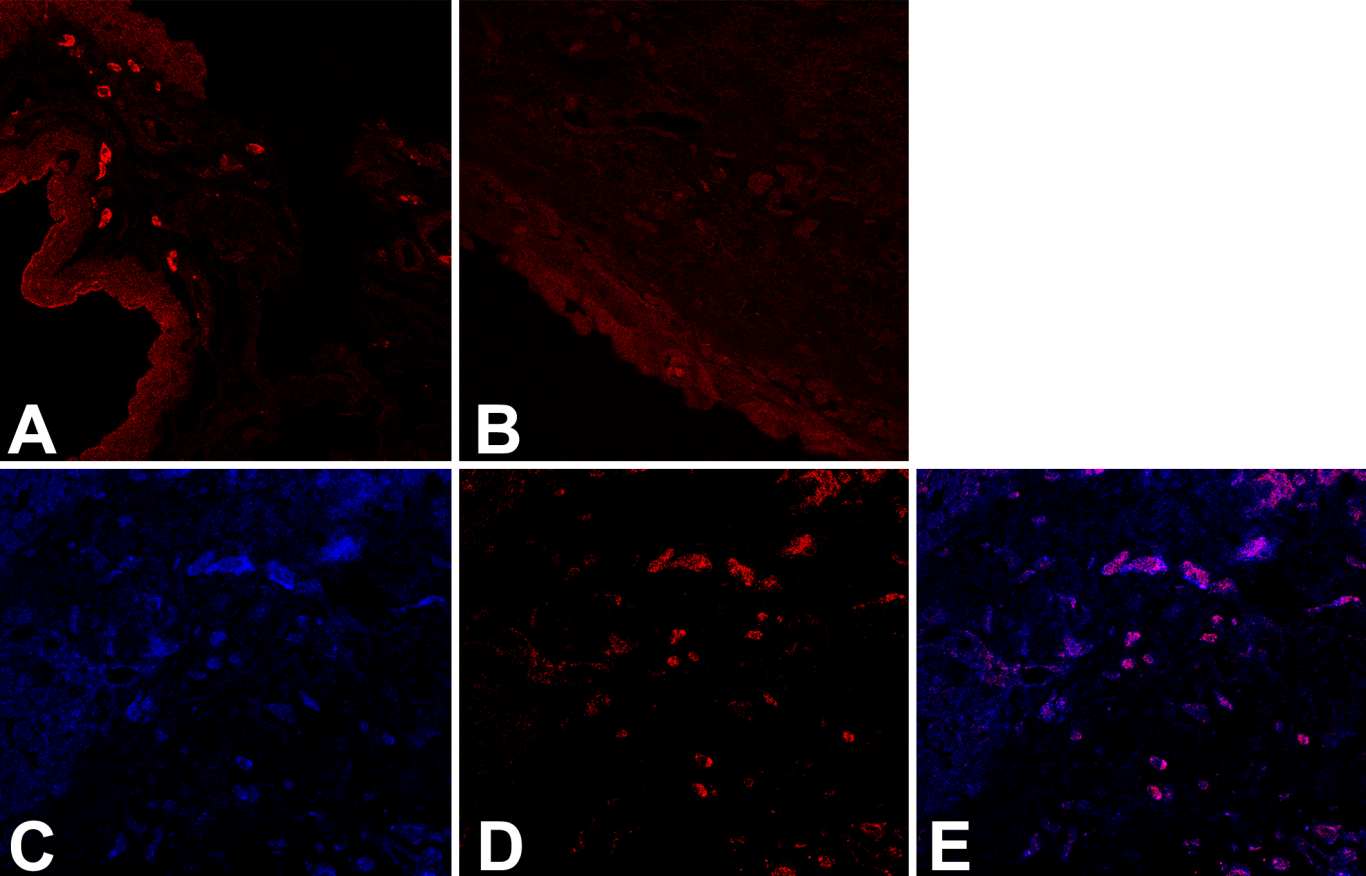
Immunolocalization of Th17 cells in OCP conjunctiva. Th17 lymphocytes were labeled with PE-conjugated anti-human IL-17 antibodies (red). Increased Th17 staining was observed in OCP (**A**) compared to healthy subjects (**B**). Double-staining for CD4 (**C**, blue) and IL17 (**D**, red) performed on four stage-III OCP samples demonstrated that 72% of CD4+ T cells were Th17 lymphocytes (Merged image **E**, violet).

All ten OCP conjunctival samples (100%) showed the presence of Th17 lymphocytes, as compared to only two of six healthy subjects (33%). Moreover, the number of Th17 lymphocytes was significantly increased in OCP compared to controls (14.9±12.8 versus 0.5±0.8, p<0.05; Figure 2 A-B). Histological analysis showed that the conjunctiva of OCP patients was infiltrated by immune cells comprised for the most part by lymphocytes, plasma cells and leukocytes (Figure 1). Since T-helper lymphocytes play a major role in the pathogenesis of OCP, the conjunctival lymphocyte infiltrate was characterized by immunostaining. CD4^+^ cells were significantly increased in OCP compared to healthy conjunctiva (14.7±6.3 versus 4.2±2.1, respectively, p<0.05). Interestingly, as shown in Figure 2 C-E, double-immunofluorescence of four stage-III OCP conjunctival samples revealed that 72% (range 57%–82%) of the CD4^+^ cells were of the Th17 type.

**Figure 3:**
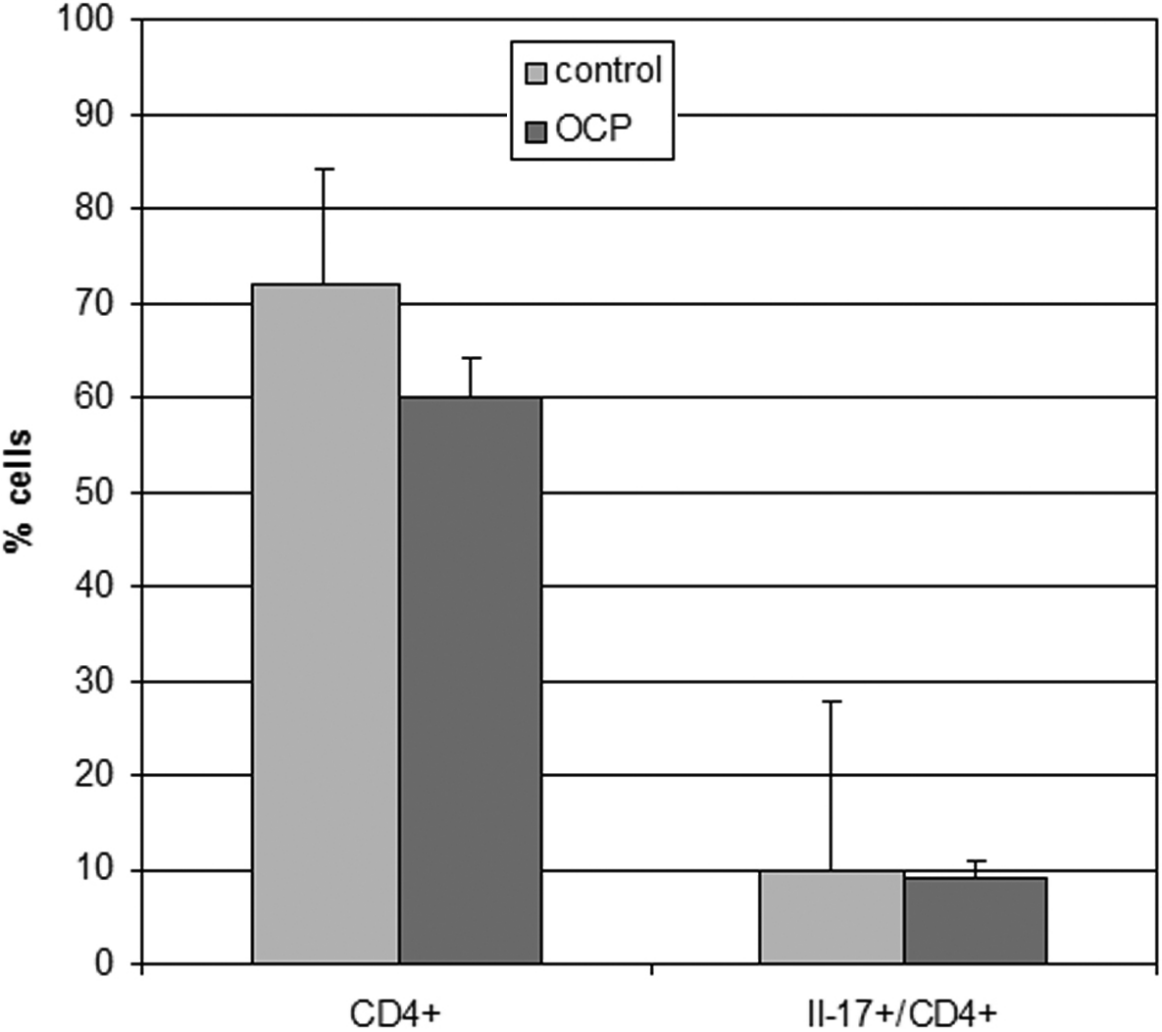
Th lymphocytes in peripheral blood. In peripheral blood, the expression of T-helper lymphocytes (CD4^+^) and Th17 (IL17^+^) was determined quantitatively with cytofluorimetry. No significant differences were observed in CD4^+^ or Th17 expression in 10 patients with OCP versus 6 healthy subjects (data are expressed as cells percentage±SD).

Since OCP is a systemic autoimmune disease, the presence of Th17 was also investigated in peripheral blood samples together with the circulating cytokine profile. Flow cytometry results showed that circulating T-helper and Th17 lymphocytes in peripheral blood were not changed in patients affected by OCP (Figure 3). The analysis of IL17-specific immunofluorescence also showed no significant difference between OCP patients and healthy subjects (MFI: 14.8±4.5 versus MFI: 17.1±3.2, ΔMIF: 2.3; p>0.05).

**Figure 4:**
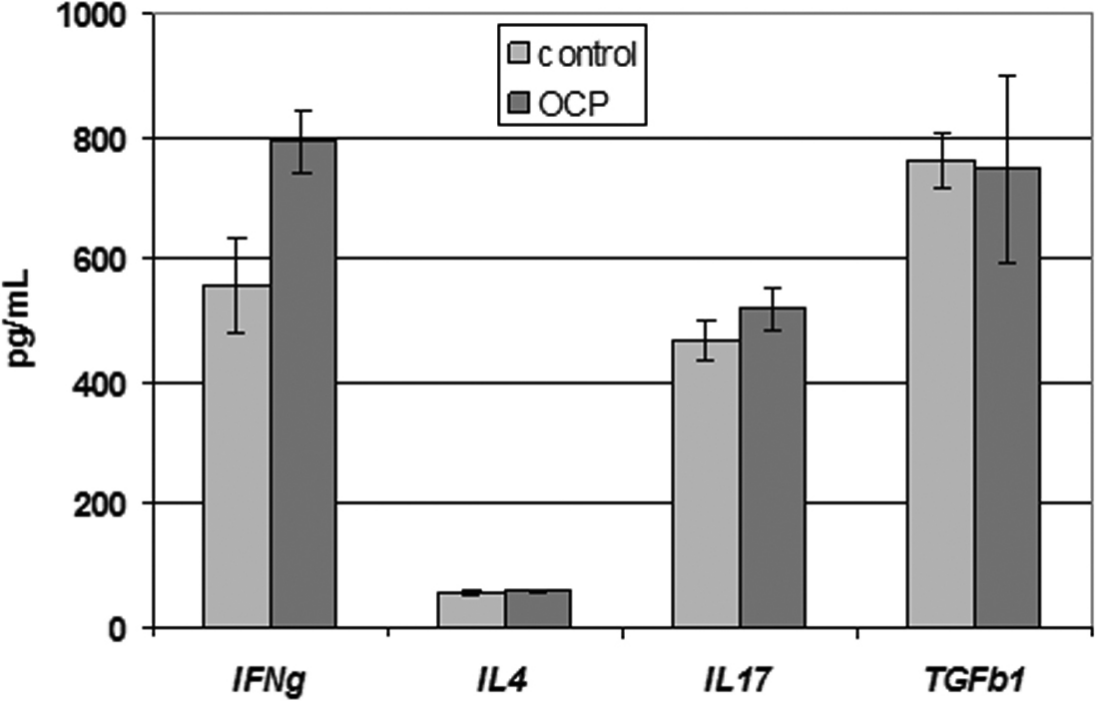
Cytokines in peripheral blood. ELISA results revealed that there was no significant difference in IL-17, IL-4 or TGF-β plasma levels in patients with OCP versus healthy subjects. A significant increase of circulating IFNγ was observed in patients with OCP. Data are expressed as mean±SD.

These results were confirmed by ELISA data regarding circulating cytokines (Figure 4). No significant differences in plasma IL-17 (518.2±75 pg/ml versus 466.1±92 pg/ml; p>0.05), IL-4 (57.7±8.4 pg/ml versus 56.8±10.8 pg/ml; p>0.05) or TGF-β (747.4±342.6 pg/ml versus 759.7±126.5 pg/ml; p>0.05) were observed between patients with OCP and healthy subjects. Conversely, a significant increase of circulating IFNγ was observed in patients with OCP (792.7±104.5 pg/ml versus 556.7±184 pg/ml; p<0.05).

## Discussion

In this study, we demonstrate that Th17 lymphocytes are present in OCP conjunctiva, and are significantly increased compared to healthy controls. Moreover, Th17 comprises the primary subtype of immune cells infiltrating the conjunctiva of OCP patients (more than 70% of CD4^+^ cells in disease stage III). These data are in line with previous results reported in other human autoimmune diseases, in which a local increase of Th17/IL-17 plays a crucial role in mucosal autoimmunity [19]. It is also worth noting that the predominant Th17 CD4^+^ subtype in our study was found in the long-standing cases of OCP (mean time from onset of disease: 4 years, range 3–9 years), suggesting that CD4^+^ Th17 mucosal infiltration could be a marker of chronic inflammatory states of the conjunctiva.

Supporting the hypothesis that Th17 lymphocytes may be responsible for a dysfunctional local immune response in the chronically inflamed OCP conjunctiva, several studies have shown that IL-17-producing-Th17 cells play a critical pathogenic role in autoimmunity, promoting organ-specific damage in several autoimmune diseases such as uveitis, Sjögren’s, MS, IBD, RA, etc [10,34-40]. In addition, the involvement of Th17 in the pathogenesis of OCP confirms previous observations on the anti-inflammatory effects of macrolides in this disease through inhibition of IL-17-induced IL-8 production [41,42].

In our study Th17 cells were also found in the circulation in both OCP and healthy subjects, where they comprise approximately 10% of CD4^+^ T lymphocytes. The apparent discrepancy that we observed between conjunctival and peripheral Th17 involvement in OCP may be explained by either a specific Th17-driven local response in the conjunctiva, or an increased Th17 homing to the ocular surface. In line with this hypothesis, Kleinscheck et al. [43] recently demonstrated that Th17 cells are imprinted for gut homing, and mediate destructive tissue inflammation in IBD. IFNγ stimulation of local Th17 trafficking may also justify the unchanged circulating number of these cells observed in the OCP patients enrolled in our study [44,45]. Our finding of increased circulating IFNγ associated with normal levels of IL17 is in line with previous reports showing that IFNγ antagonizes Th17 cell development, and allows speculating that overexpression of IFNγ in OCP patients could provoke a compensatory mechanism that ameliorates autoimmune tissue damage [46].

The findings of this study suggest a role of Th17 cells in the pathogenesis of OCP. Identification of novel immune targets such as this provide focus to the search for a more effective therapeutic strategy in OCP. Th17 has already been targeted in other autoimmune diseases and preliminary data indicate that inhibition of Th17 might cure IBD in animal models [47].
